# Supplementary material for: Long-Term and Low-Level Envelope C2V3 Stimulation by Highly Diverse Virus Isolates Leads to Frequent Development of Broad and Elite Antibody Neutralization in HIV-1-Infected Individuals
Source: Microbiol Spectr. 2022 Nov 29;10(6):e01634-22. doi: 10.1128/spectrum.01634-22 (PMC9769935; doi:10.1128/spectrum.01634-22)
Supplement: Supplemental file 1 — Tables S1 to S4 and Fig. S1 to S4. Download spectrum.01634-22-s0001.pdf, PDF file, 1.4 MB [file spectrum.01634-22-s0001.pdf]

Table S1- Characteristics of the HIV-1-infected patients.

Characteristics	2001	2009	2014
Total number of patients, n (%)	106 (28.3)	210 (56.0)	59* (15.7)
Age (years) median (IQR)	32 (26-40)	32 (28-39)	39 (36-46)
Sex, n (%)			
Female	53 (50.0)	145 (69.0)	44 (74.6)
Male	43 (40.6)	65 (31.0)	15 (25.4)
Unknown	10 (9.4)	--	--
Geographic origin, n (%):			
Angola	94 (88.7)	207 (98.6)	57 (96.6)
DRC	--	3 (1.4)	1 (1.7)
Unknown	12 (11.3)	--	1 (1.7)
HIV-1 mode of transmission, n (%):			
Heterosexual	38 (35.8)	210 (100.0)	56 (94.9)
Bisexual	4 (3.8)	--	--
IDU	1 (0.9)	--	--
Transfusion	1 (0.9)	--	--
Unknown	62 (58.5)	--	3 (5.1)

CD4+ T cell count	--	N=162	N=21
CD4+ T cell count/mm^3, median (IQR)	N/A	265 (133-448)	475 (343-569)
Plasma viral load	N=16	N=71	N=13
VL (copies/ml), median (IQR)	390,877 (209,172-704,286)	93,391 (28,222-510,579)	11,660 (380-30,060)
Undetectable, n (%)	--	--	N=9 (69.2)
Unknown, n (%)	90 (84.9)	139 (66.2)	46 (78.0)
Co-morbidities, n (%):			
Tuberculosis	--	33 (15.7)	--
HBV	--	17 (8.1)	--
TB+HBV co-infections	--	3 (1.4)	--
Other	--	48 (22.9)	--
WHO Clinical stage, n (%):			
Asymptomatic	13 (12.3)	--	--
Symptomatic intermediate	20 (18.9)	--	1 (1.7)
AIDS	11 (10.4)	--	1 (1.7)
Unknown	62 (58.5)	210 (100.0)	57 (96.6)
cART, n (%):			
cART-naïve	102 (96.2)	202 (96.2)	1 (1.7)

cART-exposed	4 (3.8)	1 (0.5)	21 (35.6)
Unknown	--	7 (3.3)	37 (62.7)

N/A, not available; DRC, Democratic Republic of Congo; IDU, intravenous drug user; VL, viral load; cART, combined antiretroviral therapy; IQR, interquartile range; HBV, Hepatitis B virus.

*53/59 HIV-1 infected patients were followed longitudinally from 2009.

Table S2- Main C2V3C3 subtypes in Angola in 2001 and 2009

**Genetic forms**	**2001** **N (%)**	**2009** **N (%)**	**P value** [a]
Pure subtypes	35/88 (39.8)	39/88 (44.3)	0.6470
Recombinant forms	53/88 (60.2)	49/88 (55.7)	
Subtype A	33/96 (34.4)	32/110 (29.1)	0.4542
Subtype C	12/96 (12.5)	30/110 (27.3)	**0.0095**
Subtype H	19/96 (19.8)	15/110 (13.6)	0.2625

[a]Fisher's exact test

Table S3- Positively selected sites in the C2, V3 and C3 regions in the four neutralization categories selected at least by two methods

**Neutralization category**	Codon*	SLAC	*p-value*	REL	PP	FEL	*p-value*	IFEL	*p-value*
**no/Weak**	293	3.673	0.101	**1.191**	1.000	1.028	0.254	**4.219**	0.059
	**335**	**3.816**	0.048	**1.612**	0.993	**0.809**	0.036	**1.376**	0.016
	336	2.793	0.139	**1.307**	1.000	3.023	0.105	**8.145**	0.020
	343	**3.820**	0.092	**1.237**	1.000	0.418	0.650	0.316	0.747
	344	1.933	0.277	**1.457**	0.997	**0.599**	0.053	0.067	0.853
	346	2.924	0.134	**1.386**	1.000	**1.587**	0.046	**0.781**	0.089
	**347**	**4.361**	0.039	**1.414**	1.000	**0.980**	0.100	0.432	0.440
	361	**3.785**	0.097	**1.267**	1.000	0.540	0.715	0.420	0.754
	362	**4.384**	0.034	**1.307**	1.000	1.015	0.140	0.344	0.562
**Cross**	318	**2.646**	0.085	0.496	0.647	**0.317**	0.053	0.000	1.000
	336	**2.582**	0.096	0.719	0.889	**0.735**	0.045	0.522	0.152
	337	**3.791**	0.041	0.721	0.887	**0.520**	0.060	0.132	0.469
	344	2.185	0.188	**0.852**	0.989	**0.599**	0.012	**1.184**	0.095
	**346**	**4.272**	0.009	**0.862**	0.991	**0.587**	0.002	**0.366**	0.066
	365	**2.034**	0.053	-0.040	0.068	**0.210**	0.038	0.000	1.000
**Broad**	335	**2.928**	0.055	0.935	0.835	**0.999**	0.047	0.000	1.000

	347	**3.383**	0.034	0.927	0.830	**1.282**	0.031	0.603	0.409
	363	**2.908**	0.068	0.583	0.609	0.978	0.274	**4.188**	0.027
**Elite**	295	2.067	0.138	**4.302**	1.000	**10.915**	0.020	8.826	0.724

*Codons selected with 10% level of significance (SLAC, FEL and IFEL) or above a Bayes Factor of 50 (REL) selected by at least 2 methods and numbered according to codon position of HIV-1 HXB2. PP, posterior probabilities. Codons selected simultaneously by SLAC, FEL and REL are bold and underlined. Bold dN-dS differences correspond to significant P-values or posterior probabilities.

Table S4- Frequency and distribution of potential N-glycosylation sites in the C2, V3 and C3 regions across neutralization categories.

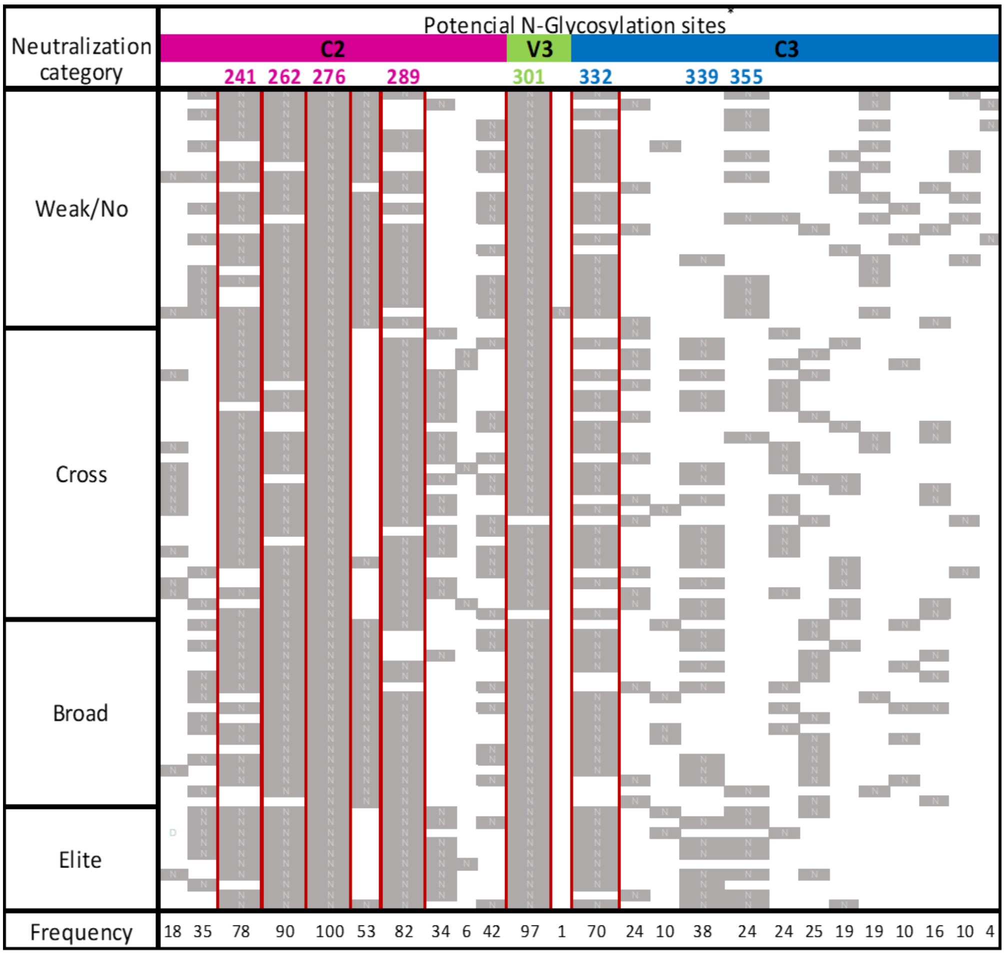

*Relevant N-glycosylation sites are highlighted and coloured according the position in C2V3C3. Higher frequency glycosylation sites are boxed in red. Sites were numbered according to the reference strain HIV-1 HXB2.

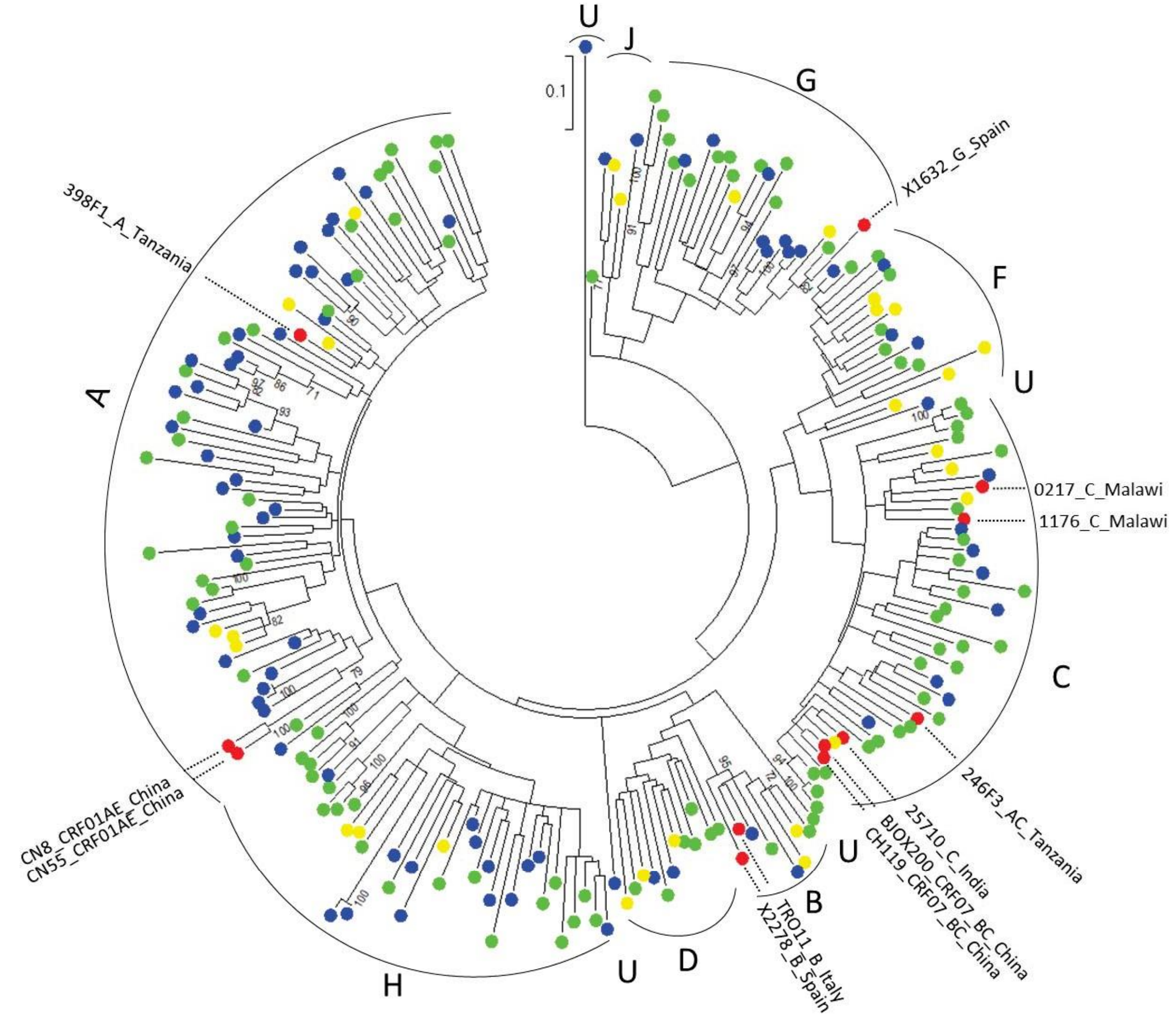

Figure S1- Phylogenetic relationship between the Angolan HIV-1 C2V3C3 sequences. Maximum likelihood phylogenetic tree of C2V3C3 region was constructed with reference sequences from all HIV-1 subtypes (yellow dots) with the 2001 (blue dots) and 2009 (green dots) Angolan sequences and the virus sequences from the indicator panel (red dots).

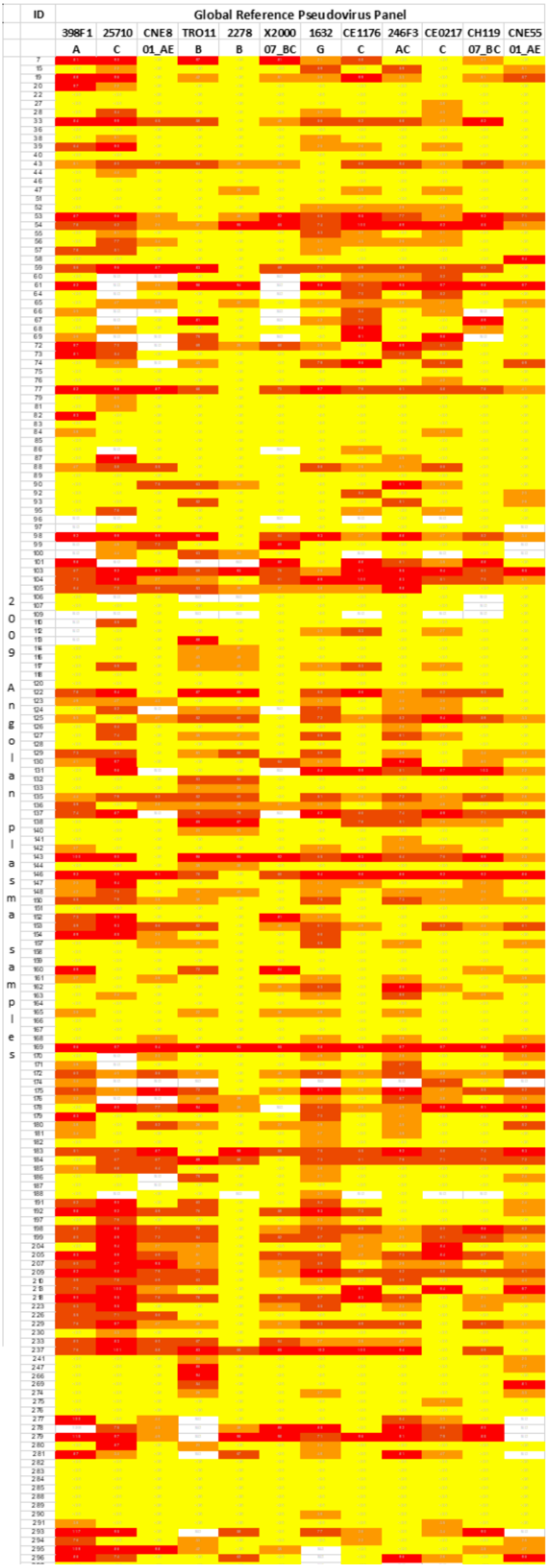
ID
Global Reference Pseudovirus Panel
398F1 A
25710 C
CNE8 01_AE
TRO11 B
2278 B
X2000 07_BC
1632 G
CE1176 C
246F3 AC
CE0217 C
CH119 07_BC
CNE55 01_AE
2009 Angolan plasma samples

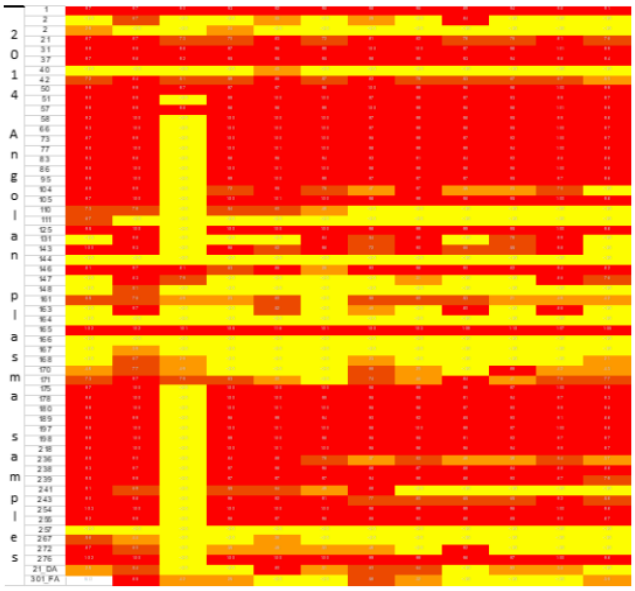

Figure S2- Heatmap showing the neutralizing activity of plasma samples from 2009 and 2014 against the 12-Env pseudotyped virus indicator panel. Percent neutralization was determined in TZM-bl cells with plasma samples diluted 1:40. White cells indicate non determined (ND) values; Yellow cells indicate <20% neutralization; orange highlighting indicates 20 to <50% neutralization; light brown highlighting indicates 50% to <80% neutralization; red highlighting indicates ≥80% neutralization. Virus subtype is indicated below the isolate common name of the Env-pseudotyped virus.

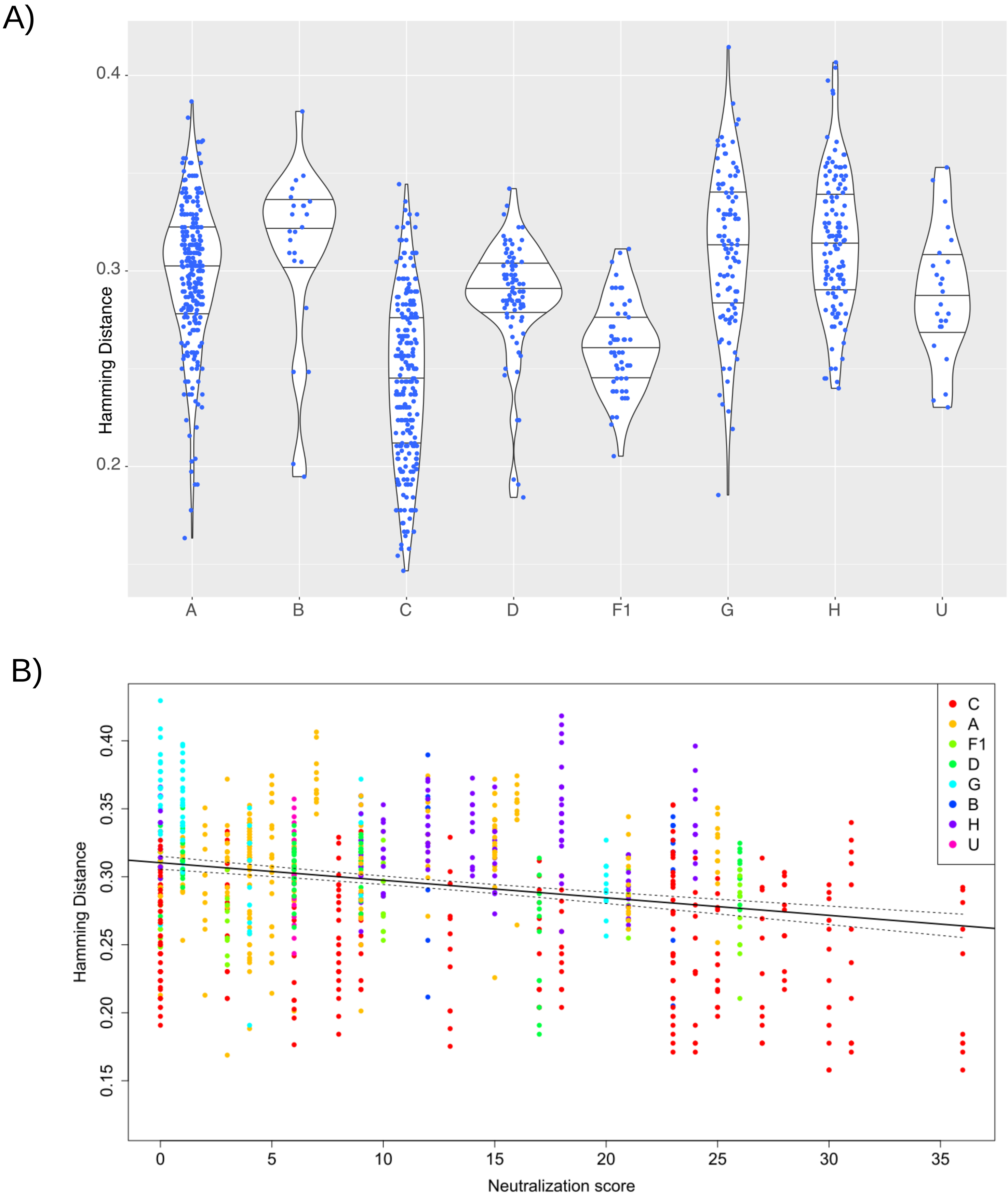

Figure S3- Impact of HIV-1 clade on antibody neutralization of the 12-virus panel. A) Violin plots showing the C2V3C3 amino acid distance of the viruses from the indicator panel to the viruses infecting the patients. The horizontal lines are the 0.25, 0.50, and 0.75 quantiles of the data in the violins. The datapoints are jittered so each one can be seen. The violin plot was made with R packages 'ape', 'Biostrings', 'DECIPHER', 'visreg', and 'ggplot2'. B) Correlation between neutralization score and C2V3C3 amino acid distance of the viruses from the indicator panel to the viruses infecting the patients. The colourful Hamming Distance across neutralization graph was done with R packages 'ape', 'Biostrings', 'DECIPHER', and 'visreg' (and 'base' graphics in R). Subtype of the patient's virus is indicated by colour, linear trend is shown with mean and 95% CI bands.

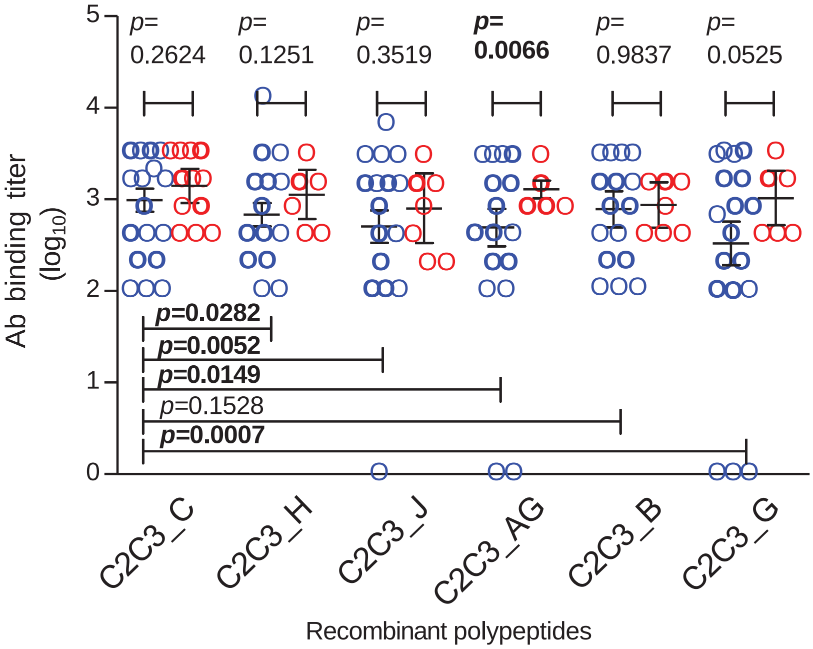

Figure S4- Antibody binding titers against the C2V3C3 recombinant polypeptides of different subtypes in patients from 2009 and 2014. Blue circles correspond to patients from 2009 and red circles to patients from 2014. Median and interquartile range are shown. P values were obtained using the Mann Whitney U test. P values <0.05 are shown in bold.
